# Lymph Node Ratio for Postoperative Staging of Laryngeal Squamous Cell Carcinoma with Lymph Node Metastasis

**DOI:** 10.1371/journal.pone.0087037

**Published:** 2014-01-27

**Authors:** Yu-Long Wang, Duan-Shu Li, Yu Wang, Zhuo-Ying Wang, Qing-Hai Ji

**Affiliations:** 1 Department of Head & Neck Surgery, Cancer Hospital, Fudan University, Shanghai, China; 2 Department of Oncology, Shanghai Medical College, Fudan University, Shanghai, China; Istituto dei tumori Fondazione Pascale, Italy

## Abstract

**Background:**

Lymph node metastasis has a significant impact on laryngeal cancer prognosis. The role of lymph node ratio (LNR, ratio of metastatic to examined nodes) in the staging of laryngeal cancer was not reported.

**Patients and Methods:**

Records of laryngeal cancer patients with lymph node involvement from Surveillance, Epidemiology, and End Results database (SEER, training set, *N* = 1963) and Fudan University Shanghai Cancer Center (FDSCC, validating set, *N* = 27) were analyzed for the prognostic value of LNR. Kaplan–Meier survival estimates, the Log-rank χ^2^ test and Cox proportional hazards model were used for univariate and multivariate analysis. Optimal LNR cutoff points were identified by X-tile.

**Results:**

Optimal LNR cutoff points classified patients into three risk groups R1 (≤0.09), R2 (0.09–0.20) and R3 (>0.20), corresponding to 5-year cause-specific survival and overall survival in SEER patients of 55.1%, 40.2%, 28.8% and 43.1%, 31.5%, 21.8%, 2-year disease free survival and disease specific survival in FDSCC patients of 74.1%, 62.5%, 50.0%, and 67.7%, 43.2%, 25.0%, respectively. R3 stratified more high risk patients than N3 with the same survival rate, and R classification clearly separated N2 patients to 3 risk groups and N1 patients to 2 risk groups (R1–2 and R3).

**Conclusions:**

R classification is a significant prognostic factor of laryngeal cancer and should be used as a complementary staging system of N classification.

## Introduction

As with other cancers of the head and neck, lymph node (LN) involvement decreases survival rates of laryngeal cancer by approximately 50%. [1] The treatment choice of laryngeal cancer depends on functional outcome, the patient’s wishes, reliability of follow-up, and general medical condition. For early-stage glottic or supraglottic cancers, surgery (partial laryngectomy through either endoscopic or open approaches) and radiotherapy seem to be equally effective. [1], [2], [3] For patients with T4a tumors, the standard approach is a total laryngectomy with ipsilateral thyroidectomy and neck dissection. [1] For managing other locally advanced, resectable glottic and supraglottic cancers, the NCCN guidelines recommended concurrent chemoradiation, surgery or induction chemotherapy as the primary care for individual choice. [1].

Postoperative radiation with or without concurrent chemotherapy were recommended by NCCN for patients with adverse features which included extracapsular nodal spread, positive margin, pT4 primary, N2 or N3 nodal disease, perineural invasion and vasular embolism. [1] Over the past 3 decades, more attention has been paid to the identification of factors that might help the surgeon to assess the precise risk of failure and benefit from more intensified therapy in individual patients. The lymph node ratio (LNR) was found to improve prognostic information in breast cancer, gastric cancer, colorectal cancer, melanoma and others. [4], [5], [6], [7] Although the prevalence of overall metastasis and occult metastasis of lymph nodes were found for 40%–75% and 26%–36% of laryngeal cancer patients, there was still no report about LNR on the survival of laryngeal cancer. [8], [9], [10].

To discuss the role of LNR for the postoperative staging of laryngeal cancer, the SEER (Surveillance, Epidemiology and End Results)-registered laryngeal cancer patients with lymph node metastasis were analyzed and the cutoff points for the LNR in defining patients as high, medium or low risk groups were also identified in current study.

## Patients and Methods

### Patients

The SEER laryngeal cancer dataset was extracted from SEER database (SEER*Stat 7.0.5). [11] Cancers were limited to the larynx, which were defined as the glottis (C32.0, vocal cord), supraglottis (C32.1, false cord, posterior surface of epiglottis, aryepiglottic fold), subglottis (C32.2), laryngeal cartilage (C32.3), overlapping lesion of larynx (C32.8) and larynx, NOS (C32.9). Histology was limited to squamous cell carcinoma (histology recode - broad groupings 8050–8090). Only laryngeal carcinomas as a single primary tumor or the first of two or more primary tumors were included. The LNR was calculated as the number of positive lymph nodes divided by the number of lymph nodes examined.

The cases with dis-concordant N classification information and the number of positive regional lymph nodes recorded in SEER database were rejected. Because the aim of current study was to identify the role of LNR staging for laryngeal cancer, the cases with unclassified T classification, M classification, grade and other variables were also enrolled in the analysis set and were defined as Tx, Mx and unknown group to avoid losing information and select bias. Finally, a total of 4183 cases of laryngeal carcinoma were collected as the analysis set, and among them 1963 cases had pathological lymph node involvement (pN+).

The FDSCC (Fudan Univesity Shanghai Cancer Center) laryngeal cancer dataset was built prospectively and recorded the layngeal cancer patients treated at Cancer Hospital, Fudan University, Shanghai, China from January, 2003 to January 2012. To avoid the bias caused by pre-operative radiotherapy and/or chemotherapy, and occult LNs metastasis, only patients primarily operated with neck dissection were enrolled in current research. The Pathologic reviews showed that 27 patients were pN+ which included 5 pN1 and 22 pN2 according to AJCC staging system. The 2-year disease specific survival (DSS, laryngeal cancer specific) and disease free survival (DFS, no local recurrence and distant metastasis) for pN+ laryngeal cancer patients were 66.3% and 51.0%, respectively.

### Ethics Statement

For SEER cases, this study was based on public use de-identified data from the U.S. SEER database and did not include interaction with human subjects or use personal identifying information. The study did not require informed consent from the SEER registried cases and the authors obtained Limited-Use Data Agreements from SEER. For FDSCC patients, written informed consent were obtained. The study was approved by the Review Board of Fudan Univesity Shanghai Cancer Center, Shanghai, China.

### Statistical Analysis

Firstly, we evaluated the prognostic value of LNR as a continuous variable, adjusting for other covariates associated with survival of 1963 SEER pN+ laryngeal cancer cases. Furtherly, we proceeded to determine the most appropriate cutoff points for categorizing LNR as high, medium, and low risk groups. Two pairs of cutoff points were identified using different methods and compared with LNR as a continuous variable to identify the optimal cutoff points. The first pair of cutoff points were identified by tertiles to split the patients into equal sized groups. [12], [13] The second pair cutoff points were calculated by X-tile using the minimum *P* values from log-rank χ^2^ statistics. [14] Finally, the prognostic significance of LNR staging was validated in FDSCC patients.

The survival rate and curves was calculated using the Kaplan–Meier method. Statistical comparisons of different factors with mortality were made with the Log-rank χ^2^ test. In multivariate analysis, forward stepwise regression analysis was carried out with a Cox proportional hazards model. Harrell’s concordance index (C-index) and AIC (Akaike information criterion) value related to the Cox regression model were analyzed to compare the predictive ability of the staging system. A smaller AIC value and a higher C-index value indicated a more desirable model for predicting outcome. A *P* value of 0.05 was considered statistically significant. All statistical analyses were carried out using SPSS software version 17.0 (SPSS Inc., Chicago, IL) and R2.14.0 software with packages (MASS and Survival).

## Results

### LNR is a Prognostic Factor of Laryngeal Cancer Survival

The clinical characteristics, 5-year cause specific survival (CSS) and overall survival (OS) estimates, and Log-rank χ^2^ test of univariate variables of the 1963 SEER patients with pN+ laryngeal cancer were shown in Table 1. Using multivariate Cox regression analysis, we found that race, radiation sequence, T classification, N classification, M classification, continuous LNR and age were all independent variables for predicting survival (Table 2).

**Table 1:** Clinicopathological characteristics, cause specific survival (CSS) and overall survival (OS) of SEER laryngeal cancer cases with pathological lymph node involvement.

Categorical variables	No. of patients	5-year CSS			5-year OS		
	(n = 1963)	Rate (%)	Log-rank χ^2^	*P* value	Rate (%)	Log-rank χ^2^	*P* value
Race			3.965	0.138		5.289	0.071
White	1480 (75.4%)	43.7			33.9		
Black	397 (20.2%)	38.8			29.7		
Other	86 (4.4%)	48.1			40.9		
Gender			2.795	0.095		1.545	0.214
Male	1526 (77.7%)	42.1			32.4		
Female	437 (22.3%)	45.9			36.8		
Year of diagnosis			0.390	0.823		0.228	0.892
1988–1994	429 (21.9%)	43.3			33.1		
1995–2001	637 (32.5%)	42.4			34.0		
2002–2008	897 (45.7%)	43.4			32.3		
Primary site			15.271	0.009		15.803	0.007
Glottis	327 (16.7%)	40.9			32.5		
Supraglottis	1285 (65.5%)	44.8			34.7		
Subglottis	36 (1.8%)	0			32.1		
Laryngeal cartilage	11 (0.6%)	32.7			24.2		
Overlapping lesion	133 (6.8%)	41.0			29.6		
Larynx, NOS	171 (8.7%)	36.0			28.7		
Histological grade			17.037	0.002		21.628	<0.001
I	89 (4.5%)	44.4			34.3		
II	937 (47.7%)	45.3			36.5		
III	783 (39.9%)	42.2			31.6		
IV	20 (1.0%)	46.1			20.0		
Unknown	134 (6.8%)	27.9			22.3		
Radiation sequence			33.328	<0.001		56.831	<0.001
No radiation	504 (25.7%)	35.4			23.7		
Pre-operative	72 (3.7%)	38.3			28.9		
Post-operative	1363 (69.4%)	45.9			37.2		
Other	24 (1.2%)	38.3			27.3		
Cancer directed surgery			26.251	<0.001		27.370	<0.001
Yes	1761 (89.7%)	44.5			34.8		
Other	202 (10.3%)	26.1			18.6		
T staging			59.303	<0.001		54.620	<0.001
T1	182 (9.3%)	57.7			46.2		
T2	526 (26.8%)	48.5			36.1		
T3	295 (15.0%)	42.1			34.7		
T4a	809 (41.2%)	39.3			31.2		
T4b	55 (2.8%)	29.6			20.3		
Tx	96 (4.9%)	24.5			17.6		
N staging			61.415	<0.001		47.574	<0.001
N1	546 (27.8%)	59.7			47.3		
N2	1313 (66.9%)	37.1			28.8		
N3	104 (5.3%)	31.9			20.6		
M staging			55.169	<0.001		45.658	<0.001
M0	1845 (94.0%)	44.1			34.3		
M1	89 (4.5%)	19.2			15.4		
Mx	29 (1.5%)	40.8			29.4		
Continuous variables	Median (range)						
Age	60 years (17–91)						
No. of LN examined	28(1–90)						
No. of positive LNs	2(1–90)						
Lymph node ratio	0.11(0.01–1.00)						

**Table 2:** Multivariate analysis of the lymph node ratio (LNR) and covariates associated with survival of laryngeal cancer cases with lymph node metastasis.

Variables	Cause specific survival		Overall survival	
	HR(95% CI)	*P* value	HR(95% CI)	*P* value
Race (White as reference)				
Black	1.212(1.041–1.412)	0.013	1.238(1.084–1.413)	0.001
Other	0.920(0.674–1.252)	0.592	0.858(0.652–1.123)	0.261
Radiation sequence (no radiation as reference)				
Pre-operative	0.846(0.606–1.182)	0.328	0.839(0.627–1.123)	0.238
Post-operative	0.694(0.599–0.805)	<0.001	0.654(0.577–0.743)	<0.001
Other	0.976(0.556–1.714)	0.932	0.952(0.574–1.579)	0.848
T classification (T1 as reference)				
T2	1.308(1.013–1.689)	0.040	1.277(1.033–1.577)	0.024
T3	1.740(1.316–2.301)	<0.001	1.540(1.216–1.951)	<0.001
T4a	1.856(1.450–2.375)	<0.001	1.630(1.327–2.002)	<0.001
T4b	2.365(1.574–3.552)	<0.001	2.157(1.526–3.048)	<0.001
Tx	1.963(1.285–3.001)	0.002	1.725(1.186–2.511)	0.004
N classification (N1 as reference)				
N2	1.948(1.668–2.274)	<0.001	1.642(1.445–1.865)	<0.001
N3	1.806(1.358–2.402)	<0.001	1.668(1.309–2.127)	<0.001
M classification (M0 as reference)				
M1	1.700(1.194–2.420)	0.003	1.579(1.137–2.195)	0.006
MX	0.722(0.386–1.349)	0.307	0.864(0.510–1.466)	0.588
LNR as continuous variable	2.299(1.905–2.775)	<0.001	1.810(1.532–2.138)	<0.001
Age	1.013(1.006–1.019)	<0.001	1.020(1.014–1.025)	<0.001

### Cutoff Points Identification of LNR

To stratify the patients with lymph node metastasis as high, medium and low risk groups associated with CSS, the upper and lower tertiles of continuous LNR that corresponded to 0.06 and 0.23 were defined as the first pair of cutoff points. The X-tile, which can control the inflated type I error problem and minimize the loss of information due to multiple testing through cross-validation, identified 0.09/0.20 as the second pair of cutoff points. [6], [14] The SEER cases with lymph node metastasis were stratified as high, medium and low risk groups according to the two pairs of cutoff points identified above. The case numbers, the 5-year CSS and 5-year OS of the different risk groups were summarized in Table 3. To compare the predictive ability of the categorical LNR and the continuous LNR, the C-index and AIC value of the Cox regression model (Table 2) with substitution of the continuous LNR with the categorical LNR were calculated. As listed in Table 3, the models of categorical LNRs defined by cutoff points 0.09/0.20 showed superior predictive ability to that of the continuous LNR and another categorical LNR, with the lowest AIC value and highest C-index value associated with the Cox regression model. The SEER laryngeal cancer patients with lymph node metastasis were classified as R1 (LNR ≤0.09), R2 (LNR 0.10–0.20) and R3 (LNR >0.20) three risk groups (R classification).

**Table 3:** Univariate and multivariate analysis of the categorical and continuous LNR with cause-specific survival (CSS) and overall survival (OS) of SEER laryngeal cancer patients with lymph node metastasis.

LNRclassification	CaseNo.	5-yearCSS(%)	Log-rankχ2 (*P* )	Multivariate analysis of CSS†			5-yearOS(%)	Log-rankχ2 (*P* )	Multivariate analysis of OS†		
				HR (95% CI)	C-index	AIC			HR (95% CIs)	C-index	AIC
Continuous LNR				2.299(1.905–2.775)	0.658	14037.85			1.810(1.532–2.138)	0.642	18430.78
Cutpoints 0.06/0.23			109.197		0.658	14042.83		87.729		0.642	18432.54
R1: 0–0.06	637	56.3	(<0.001)	Reference			43.9	(<0.001)	Reference		
R2: 0.06–0.23	737	42.4		1.270(1.074–1.502)			33.4		1.233(1.070–1.421)		
R3: >0.23	589	29.4		1.928(1.635–2.273)			22.2		1.624(1.409–1.871)		
Cutpoints 0.09/0.20			125.228		0.661	14027.14		94.673		0.645	18424.27
R1: 0–0.09	860	55.1	(<0.001)	Reference			43.1	(<0.001)	Reference		
R2: 0.09–0.20	458	40.2		1.421(1.202–1.680)			31.5		1.308(1.133–1.509)		
R3: >0.20	645	28.8		1.962(1.695–2.271)			21.8		1.607(1.416–1.825)		

† The multivariate analysis was adjusted using the same Cox regression model at Table 2.

### Selecting High Risk Patients by R classification

**Figure 1:**
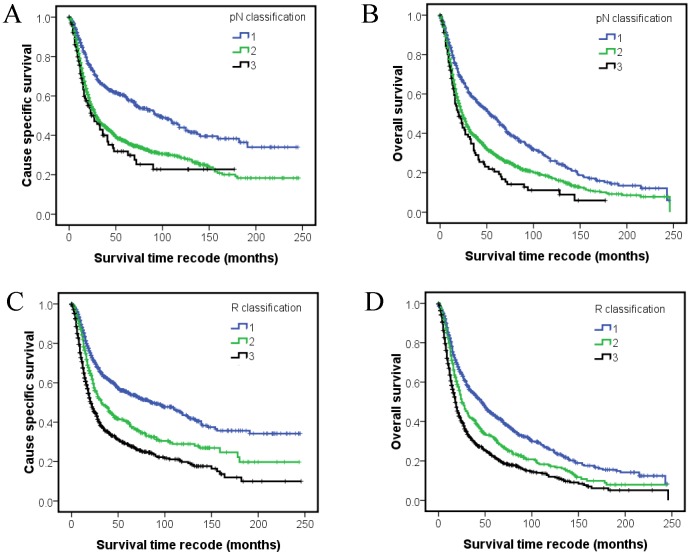
Kaplan–Meier survival estimates according to pN classification and R classification of SEER laryngeal cancer patients with lymph node metastasis: cause-specific survival (A) and overall survival (B) of the SEER set with different pN classification; cause-specific survival (C) and overall survival (D) of the SEER set with different R classification.

N classification was widely used for postoperative staging of lymph node of laryngeal cancer, while the cause specific survival curves of N3 crossed with N2 after 150 months follow-up (Figure 1A). N1, N2 and N3 accounted for 27.8%, 66.9% and 5.3% of all pN+ patients (Table 1). Compared with pN classification, the survival curves of individual R classification separated clearly even after 20 years follow-up (Figure 1C and 1D). The R classification also showed homogenous patients grouping which stratified the patients to 43.8% (R1), 23.3% (R2) and 32.9% (R3) of all pN+ patients (Table 3). The 5 and 10-year CSS and OS of N3 patients were and 31.9%, 22.7% and 20.6%, 11.1%, individually. The 5 and 10-year CSS and OS of R3 patients were and 28.8%, 19.8% and 21.8%, 12.0%, respectively. R3 stratified more high risk patients than N3 with the same survival rate.

### R classification Stratify Individual N Patients to Different Risk Groups

**Figure 2:**
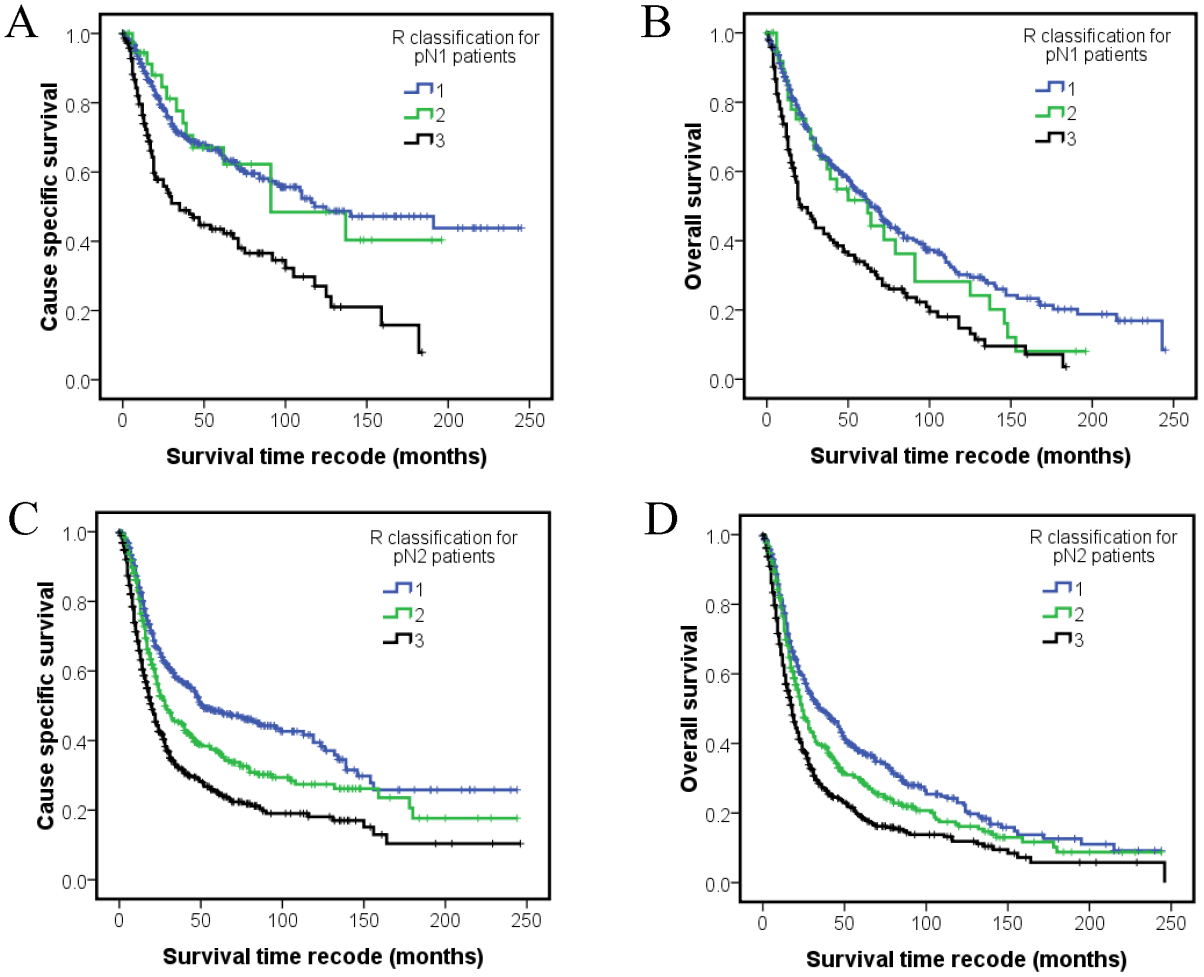
Kaplan–Meier survival estimates of different R classification patients of individual N classification. The cause-specific survival (A) and overall survival (B) of individual R classification for pN1 patients; cause-specific survival (C) and overall survival (D) of individual R classification for pN2 patients.

To further analyze the role of R classification for stratify patients to different risk groups, the R classification were defined for patients with individual N classification. For pN1 patients, the 5-year CSS and OS of R1 (*N* = 361), R2 (*N* = 40), R3 (*N* = 145) groups were 65.3%, 67.0%, 43.5% (Log-rank χ^2^ 32.117, *P*<0.001) and 52.7%, 51.7%, 33.0% (Log-rank χ^2^ 27.654, *P*<0.001), respectively (Figure 2A and 2B). For pN2 patients, the 5-year CSS and OS of R1 (*N* = 468), R2 (*N = *400), R3 (*N* = 445) groups were 48.5%, 37.1%, 25.1% (Log-rank χ^2^ 63.523, P<0.001) and 37.6%, 29.6%, 18.9% (Log-rank χ^2^ 45.328, *P*<0.001), respectively (Figure 2C and 2D). No significant differences of CSS (Log-rank χ^2^ 5.493, *P* = 0.064) and OS (Log-rank χ^2^ 2.713, *P* = 0.258) were observed for individual R classification group of pN3 patients (*N = *104). R classification clearly separated N2 patients to 3 risk groups and N1 patients to 2 risk groups (R1–2 and R3). The survival of R1 patients of N2 classification is better than the survival of R3 patients of N1 classification. All these results supported that R classification can stratify the patients to different risk groups and complement N classification.

### Validation of R Classification in FDSCC Patients

To validate R classification in the FDSCC patient set, 27 pN+ cases were analyzed and the median number of LNs examined, positive LNs and LNR were 38 (range, 8–123), 2 (range, 1–28) and 0.09 (range, 0.01–0.70), respectively. The 2-year DSS and DFS of R1 (*N* = 13), R2 (*N* = 10), R3 (*N* = 4) patients were 74.1%, 62.5%, 50.0%, and 67.7%, 43.2%, 25.0%, respectively. The 2-year DSS and DFS of N1 (*N* = 5) and N2 (*N* = 22) patients were 50.0%, 69.5% and 50% and 47.6%, respectively. Although no statistical significance were found for survival difference of individual R classification and N classification due to less patients number, the R classification stratify patients to 3 risk groups clearly.

## Discussion

When surgery was selected as the primary management of laryngeal cancer, more attention should be paid to identify factors that might help the surgeon to assess the precise risk of failure. The main prognosticators included T classification, quality of surgical resection, positive margins, two or more positive lymph nodes, largest node >3 centimeters in diameter, perineural invasion, and in some studies, age and gender. [15], [16] In 2004, level I evidence was established for the postoperative chemoradiotherapy treatment of patients with selected high risk locally advanced head and neck cancers, with the publication of the results of two trials conducted in EROTC and RTOG. Extracapsular extension and/or microscopically involved surgical margins were the only risk factors for which the impact of chemotherapy enhanced radiation therapy was significant in both trials. [17] Our current results identified that lymph node ratio is as independent risk factors for the postoperative staging of laryngeal cancer. The R classification defined by the cutoff points 0.09/0.20 can clearly stratify patients to different risk groups and complement the pN classification. The R classification should be incorporated into the risk factors mentioned above to stratify patients and assess the treatment benefits. In this exciting and chaotic period in which new chemotherapy agents, new paradigms of treatment, new surgical and radiation technology, and new prognostic factors are rapidly becoming available, a multidisciplinary and collaborative approach for each patient at the start of decision making and planning is a necessity and the absolute standard of medical treatment for excellent patient care. [18].

The significant decrease in the number of laryngeal cancer surgical cases and changing use of new drugs and radiation techniques in the chemoradiation era prevent a clear and accurate analysis of risks of postoperative failure based on single institution’s experience. SEER data are abstracted prospectively from registries comprising 26% of U.S. population, which is considered representative of the entire population, and the selection biases, recall biases, treatment fads, influence of loss to follow-up and other oversights associated with single institution’s research were minimized. [11], [19] Current studies use the largest series (SEER) of operated pN+ laryngeal cancer cases to analysis the role of R classification for staging of laryngeal caner and validate the R classification in an independent dataset (FDSCC). The complementary data collection system and the cross-validation of SEER and FDSCC dataset reinforce the conclusion. While limitations still exist for current research, such as the inter-institution differences in patient management, unrecorded details of pathologic reports and medical management covariates of SEER dataset, the less patient number of FDSCC dataset. Integrating R classification with other risk factors to analysis the pooled data of inter-institutional clinical trials will achieve a definitive result of the postoperative staging of laryngeal cancer.

In conclusion, we clearly identify that lymph node ratio was an independent prognostic factor of laryngeal cancer and R classification (LNR 0–0.09, LNR 0.09–0.20 and LNR >0.20)defines laryngeal cancer mortality adequately. R staging is a complementary staging system for N classification. R staging based stratification of patients for postoperative therapy and clinical trials deserved for further research.
